# Effect of Structure and Hydrogen on the Short-Term Creep of Titanium Ti-2.9Al-4.5V-4.8Mo Alloy

**DOI:** 10.3390/ma15113905

**Published:** 2022-05-30

**Authors:** Galina Grabovetskaya, Ivan Mishin, Ekaterina Stepanova, Olga Zabudchenko

**Affiliations:** 1 Federal State Institution of Science, Institute of Strength Physics and Materials Science of the Siberian Branch of the Russian Academy of Sciences, 634055 Tomsk, Russia; grabg@ispms.tsc.ru (G.G.); mishinv1@yandex.ru (I.M.); lekalune@mail.ru (O.Z.); 2 Division for Experimental Physics, National Research Tomsk Polytechnic University, 634050 Tomsk, Russia

**Keywords:** titanium alloy, ultrafine-grained structure, creep, hydrogen

## Abstract

In this paper, the effect of hydrogenation, in the amount of 0.15 wt.%, on the short-term creep of a titanium Ti-2.9Al-4.5V-4.8Mo alloy in fine-grained (FG) and ultrafine-grained (UFG) states is studied at 723 K. The UFG structure was formed by the method of pressing with the change of the deformation axis and gradual temperature decrease. Creep tests are performed under conditions of uniaxial tension at a constant load for the creep rates at an interval of (10^−7^ ÷ 10^−6^) s^−1^. The UFG alloy’s resistance to creep under the investigated conditions is revealed to be substantially lower than in the FG state. When hydrogen presents in the alloy in a solid solution, a 1.3–2.5-fold rise in the value of the steady-state creep rate for the hydrogenated FG and UFG alloys is observed. The creep of the non-hydrogenated FG and UFG alloys is described by the creep power law. The presence of dissolved hydrogen leads to a violation of the creep power law. The values of stress sensitivity indices, steady-state creep rate, and effective creep activation energy are determined. The relationships between the hydrogenation, structure, and creep mechanisms of the alloy at the steady-state are discussed.

## 1. Introduction

Titanium alloys have high specific strength and corrosion resistance under atmospheric conditions and with a number of strong chemicals. This unique combination of properties allows for using titanium alloys in various branches of industries and medicine [1,2,3,4,5,6,7]. Titanium two-phase (α + β type) Ti-2.9Al-4.5V-4.8Mo alloy is widely used for the manufacturing of implants and medical instruments [2,8], as well as fastening elements in the aerospace industry and shipbuilding [2,9]. The alloy has a high corrosion resistance and good ductility at room temperature (6–8%), and it can be strongly strengthened during heat treatment [8,10]. The development of aerospace and automotive technology requires a further increase in the specific strength of titanium alloys. A promising method for increasing the specific strength of the Ti-2.9Al-4.5V-4.8Mo alloy, as well as other titanium alloys, is the formation of an ultrafine-grained (UFG) structure by methods of severe plastic deformation [11,12,13]. Thus, the formation of an UFG structure in the Ti-2.9Al-4.5V-4.8Mo alloy, with an average grain–subgrain structure element size of 0.2–0.4 μm, allows for increasing its strength characteristics by 30–40% (up to 1400 MPa), while maintaining ductility at 12–13% [14]. At the same time, it is known that a decrease in grain size leads to an increase in the absorption of hydrogen by titanium and its alloys [15]. In turn, hydrogen, depending on the concentration and deformation conditions, can have a plasticizing [15,16] or embrittling [16,17,18] effect on titanium and its alloys. The plasticizing effect of hydrogen is often used in the molding of products from titanium alloys [16,19].

The development of hydrogen embrittlement at low temperatures in materials such as titanium and its alloys is primarily associated with the formation of hydrides [16,17]. The surface energy of titanium (1000–1200 erg/cm^2^ [17]) is more than three times higher than the surface energy of titanium hydride (300 erg/cm^2^ [17]). Therefore, fractures along the titanium–hydride surface occur at a lower stress than along the titanium–titanium surface. At elevated temperatures (above 623 K), hydrides completely dissolve in titanium and its alloys. In this case, dissolved hydrogen can lead to the embrittlement of titanium alloys during operation, even if its concentration does not exceed the maximum permissible value. At present, despite systematic studies, there is no consensus in the literature data regarding the mechanisms of the development of hydrogen embrittlement in metallic materials caused by the presence of dissolved hydrogen. The authors of [16,17] associated the embrittlement effect of dissolved hydrogen with its ability to redistribute over the metal bulk under the action of elastic stress fields, forming pores and clusters in the most stressed regions. In [20,21], simple and complicated hydrogen-vacancy complexes, which are formed during hydrogenation and can be the nuclei of microdamages, were considered the cause of hydrogen embrittlement due to dissolved hydrogen. Currently, the theory of hydrogen-enhanced decohesion (HEDE) [22] and mechanism of hydrogen-enhanced localized plasticity (HELP) [23] are the most popular theories explaining the embrittling impact of dissolved hydrogen on metallic materials. According to HEDE theory [22], dissolved hydrogen, being at the grain boundaries, weakens interatomic bonds, thereby reducing the energy of crack formations. Materials in the UFG state have a greater extension of grain boundaries, compared to the fine- (FG) and coarse-grained (CG) states. Therefore, it can be expected that, at the same concentration of dissolved hydrogen, UFG titanium alloys will have greater resistance to hydrogen embrittlement, in comparison with the FG and CG states. On the other hand, in accordance with the HELP mechanism [23,24,25,26], hydrogen that has dissolved in the metal shields the interaction of dislocations, which results in a drop in the energy of the dislocation nucleation, as well as a rise in its mobility. This promotes a dislocation cluster formation in regions with higher hydrogen concentration (in the zones of stress localization). The annihilation of dislocation clusters will result in microcavity and crack formation. According to the model [27], the rate of dislocation annihilation in UFG metals, due to the high density of high-angle boundaries and depending on temperature and stress, can be higher than that of the FG and CG metals. Therefore, in the presence of hydrogen, the rate of formation of microcavities and cracks in UFG metal can be higher, as compared to the FG and CG metals. The hydrogen embrittlement of metallic materials caused by the dissolved hydrogen manifests itself at the low strain rates characteristic of creep. Such strain rates usually occur in the continuous service of products made of titanium alloys, as well as in the molding of parts made of titanium alloys via the creep method [19,28]. However, there are few studies in the literature that are devoted to the effect of hydrogen on the regularities of creep of titanium alloys in the UFG state. The data that are available in the literature on the effect of hydrogen on the creep of titanium alloys in the UFG state are contradictory and refer to alloys of the Ti-Al-V system [29,30,31]. Thus, it was shown in [29,30] that dissolved hydrogen reduces the steady-state creep rate of UFG alloys of the Ti-Al-V system at room and elevated temperatures. However, it was found in [31] that, at elevated temperatures, dissolved hydrogen neither affects nor increases the steady-state creep rate of alloys of the Ti-Al-V system. In addition, the content of the β phase in alloys of the Ti-Al-V system, as a rule, does not exceed 10% volume. Hydrogen in titanium alloys is contained in the β phase. Therefore, the effect of hydrogen on the creep of alloys with a high content of the β phase, which includes the Ti-2.9Al-4.5V-4.8Mo alloy, may be different, compared to alloys of the Ti-Al-V system.

The purpose of this work is a comparative study of the effect of dissolved hydrogen on the regularities of short-term creep of the Ti-2.9Al-4.5V-4.8Mo alloy in the FG and UFG states at a temperature of 723 K, as well as the rates typical for the molding of products made of titanium alloys via the creep method.

## 2. Materials and Methods

### 2.1. Material

The commercial FG (α + β) titanium alloy of the Ti-Al-V-Mo system has the following elemental composition (wt%): 2.9Al + 4.5V + 4.8Mo + 0.03Zr + 0.05Si + 0.06Fe + ≤0.1C + 0.15O + 0.05N + 0.002H; the balance, Ti, was used as an initial material for the study (hereinafter, the FG Ti-2.9Al-4.5V-4.8Mo alloy).

The FG Ti-2.9Al-4.5V-4.8Mo alloy was hydrogenated to the concentration of ~0.15% (hereinafter, the hydrogen concentration is indicated as the weight per cent), using the automated complex gas reaction controller at a temperature of 873 K and hydrogen pressure of 1 atm (hereinafter, this material is the FG Ti-2.9Al-4.5V-4.8Mo-0.15H alloy). The absolute hydrogen concentration in alloy billets was measured by melting in argon media using a RHEN 602 gas analyzer.

The method of pressing with the change of the deformation axis and gradual temperature decrease was applied, in order to obtain the UFG structure in both types of FG titanium alloys. Previously, it was established [32] that the presence of hydrogen in a solid solution reduces the value of deformation required for the formation of an UFG structure in hydrogenated material, compared to a non-hydrogenated one. Therefore, to obtain a UFG structure with similar dimensional characteristics, pressing of the FG Ti-2.9Al-4.5V-4.8Mo and Ti-2.9Al-4.5V-4.8Mo-0.15H alloys was performed using two different Modes. Firstly, the FG Ti-2.9Al-4.5V-4.8Mo alloy was pressed in five cycles, with a gradual temperature decrease in the range of 1023–773 K (hereinafter, the UFG Ti-2.9Al-4.5V-4.8Mo alloy). Before pressing, the alloy was quenched in water from a temperature of 1068 K. One pressing cycle included three compressions at a given temperature. Deformation after a single compression was ~50%. The pressing rate was ~10^−3^ s^−1^. Secondly, formation of the UFG state in FG Ti-2.9Al-4.5V-4.8Mo-0.15H alloy was carried out using the following scheme: quenching from 873 K and subsequent pressing, consisting of two cycles at temperatures of 873 and 773 K (hereinafter, the UFG Ti-2.9Al-4.5V-4.8Mo-0.15H alloy).

It was found in [33,34,35,36] that the UFG state of titanium and its alloys (formed using plastic deformation) were nonequilibrium. During holding at elevated temperatures, recovery processes, grain growth, and phase transformations developed in such structures. Consequently, the results of creep studies at elevated temperatures can depend on the indicated temperature changes in the UFG structure. Therefore, in this work, the UFG and FG structures of the Ti-2.9Al-4.5V-4.8Mo and Ti-2.9Al-4.5V-4.8Mo-0.15H alloys were stabilized before tensile and creep tests by annealing at a temperature of 673 K for 30 h.

### 2.2. Material Characterization

The structure of the alloys was investigated with the AXIOVERT-200MAT optical and JEM-2100 transmission electron (accelerating voltage 200 kV) microscopes. The dimensions of the structural elements of the alloys were retrieved from the corresponding images of the microstructure by the secant method. The sample for the FG structure was at least 70 elements; for the UFG structure, it was at least 180 elements.

The phase composition, lattices parameters, and microdeformations of the phase crystal lattices were determined using a Shimadzu XRD-6000 diffractometer, with a Cu-K_α_ radiation source and at a voltage of 40 kV. The preset time was 1 s, step size was 0.2 deg, and angle range was (30–90) deg. The Shimadzu XRD-6000 diffractometer was equipped with the PowderCell software package. The qualitative phase composition of the alloy studied was determined using the ICDD PDF4+ database. The calculation of the volume content of the phases was carried out using the PowderCell program via the Rietveld method [37]. The value of internal elastic stresses (σ) was assessed by the value of microdistortions of the crystal lattice (ε) using the formula [38]:

(1) σ=E⋅ε/μ,

where *E* is Young’s modulus (for the α phase, *E* = 110 GPa; for the β phase, *E* = 51 GPa [33]); μ is Poisson’s ratio (0.32 [38]).

The dislocation density was determined from the diffraction maxima broadening at their half-height using the Cauchy approximation. To separate the contributions of the coherent scattering regions and lattice microdeformation to the broadening of diffraction maxima for the bcc lattice of the β phase, we used the (110)_β_ and (220)_β_ plane diffraction peaks. For the first approximation, the density of dislocations (ρ) randomly located in the grain in the bcc lattice can be calculated from the formula [39]:

(2) $$\rho =\beta_{\varepsilon }^{2}/9b^{2},$$

where β_ε_ = (β_110_ + β_220_)/2 is the diffraction maximum broadening, due to the lattice microdeformation; *b* is the Burgers vector for the β phase of titanium (2.86·10^−10^ m [40]).

For the hcp lattice, it is difficult to obtain the maxima of the second order. Therefore, we used the diffraction maxima of the (101)_α_ and (110)_α_ planes. The density of dislocations randomly located in the grain in the hcp lattice was calculated from the formula [39]:

(3) $$\rho =\sqrt{\pi }\beta_{\varepsilon }^{2}/b^{2},$$

where β_ε_ = (β_101_ + β_110_)/2 is the diffraction maximum broadening, due to the lattice microdeformation; *b* is the Burgers vector for the α phase of titanium (2.95·10^−10^ m [40]).

### 2.3. Mechanical Tests

Tensile and creep tests were conducted in a vacuum of 10^−2^ Pa using the universal PV-3012M machine. The installation had a system for automatic recording of the stress–strain flow curve in load-time coordinates. The initial tension rate under tests was 6.9·10^−3^ s^−1^. The installation provided isothermal test conditions. During the tests, the temperature drift was within ±0.5 K.

Preliminary tensile tests in the temperature range of 293–873 K have shown that the values of the strength characteristics of the UFG alloys are significantly higher, compared to the FG state at temperatures 293–673 K. However, the rate of softening of UFG alloys was higher with increasing test temperature in the studied range, in comparison to the FG state. At a temperature of 873 K, the strength characteristics of the UFG alloys become noticeably lower than in the FG state. At a temperature of 723 K, the strength characteristics of the alloy in FG and UFG were close. In addition, at a temperature of 723 K, hydrogen is in a solid solution and, during long-term annealing at this temperature in vacuum, its concentration in the alloy does not change. At a temperature of 773 K and above, during creep tests in a vacuum, degassing of hydrogen from the alloy can occur. At temperatures of 673 K and below, hydrides can form in titanium alloys. Therefore, creep tests were carried out at a temperature of 723 K (below the temperature of the last pressing of alloys (773 K)).

Creep tests were performed under conditions of uniaxial tension and constant load for creep rates in the interval (10^−7^ ÷ 10^−6^) s^−1^. The choice of indicated creep rates was caused by their use in the molding of products of titanium alloys by creep methods. The elongation of the tested specimens was measured with an optical cathetometer (KM-6 type), with an accuracy of ± 5μm. The method of temperature rise during the temperature change was applied to define the value of effective creep activation energy (*Q_c_*) [41]. Calculation of the *Q_c_* value was carried out according to the formula [41]:

(4) $$Q_{c}=R\ln({\dot{\varepsilon }}_{2}/{\dot{\varepsilon }}_{1})/(1/T_{1}-1/T_{2})$$

where ${\dot{\varepsilon }}_{1}$ and ${\dot{\varepsilon }}_{2}$ are the minimum creep rates, before and after the temperature change, and *R* is the universal gas constant; *T*_1_ and *T*_2_ are absolute temperatures.

Samples used for the above-mentioned tests have the form of a double blade and dimensions of the working part of 5 × 1.7 × 0.7 mm^3^. In tensile and creep tests, at least three specimens were used in each experiment. The scattering in the values determined from the tension and creep curves did not exceed 5%.

After the creep tests, the deformation relief of the working part of the sample and fracture surfaces were investigated using a Quanta 200 3D scanning electron microscope with 30 kV voltage.

## 3. Results

### 3.1. Microstructure and Phase Composition

**Figure 1:**
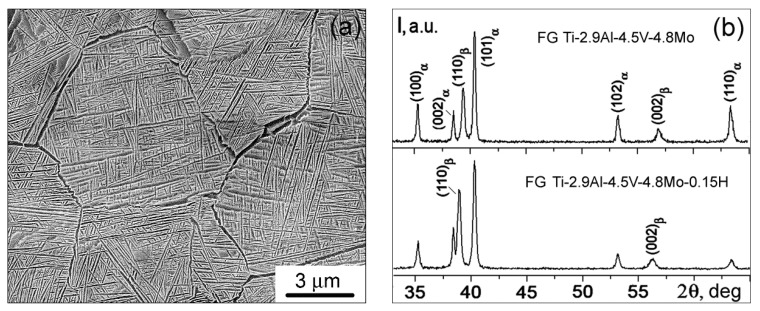
Microstructure (**a**) and diffraction patterns (**b**) of the FG titanium alloys.

In the initial FG state, the investigated Ti-2.9Al-4.5V-4.8Mo alloy has a lamellar structure with a grain size of the initial β phase d ~9 μm (Figure 1a). The alloy is two-phase and contains both the α and β phases of titanium (Figure 1b, curve 1). The volume fraction of the β phase is (20 ± 1) vol.%. Hydrogenation to ~0.15% does not change the morphology of the alloy structure. Simultaneously, the volume fraction of the β phase increases to (27 ± 1) vol. (Figure 1b, curve 2).

**Figure 2:**
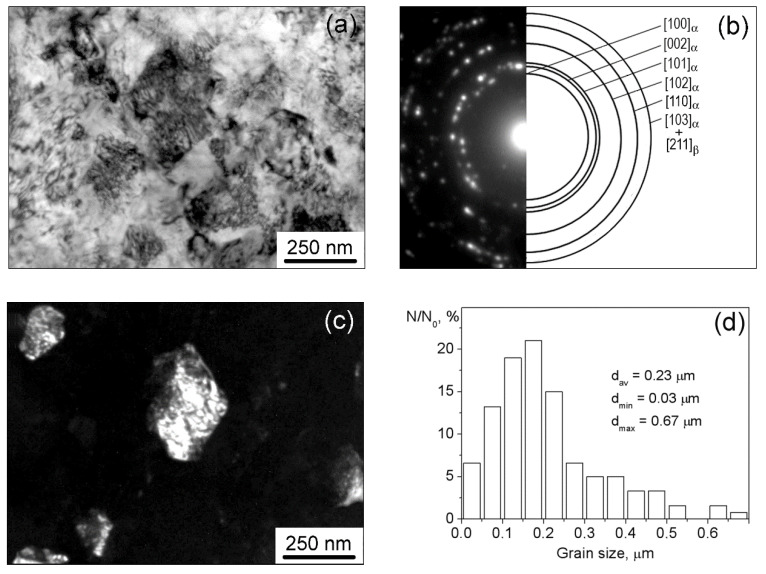
TEM images of the UFG Ti-2.9Al-4.5V-4.8Mo alloy: (**a**) bright-field image and (**b**) corresponding SAED; (**c**) dark-field image in the reflection of (002)_α_ type; (**d**) size distribution of grain–subgrain elements.

The electron microscopic image of the UFG structure of the Ti-2.9Al-4.5V-4.8Mo alloy is represented in Figure 2a. A significant number of reflections evenly distributed over a circle in the SAED (Figure 2b) and obtained for an area of 1.4 μm^2^ indicates the presence of a large number of elements in the structure unit volume and their substantial misorientation. Detailed studies of the dark-field images of the structure (Figure 2c) showed that the UFG grain–subgrain structure was formed in the Ti-2.9Al-4.5V-4.8Mo alloy as a result of pressing (Figure 2d). The dimensions of the structure elements varied mainly in a range from 0.05 to 0.25 μm (Figure 2d). The average size of the grain–subgrain structure elements (d_av_) retrieved from the dark-field image was equal to (0.23 ± 0.14) μm. In this case, a lamellar structure with a transverse plate size of 10–30 nm was observed in some of the elements.

**Figure 3:**
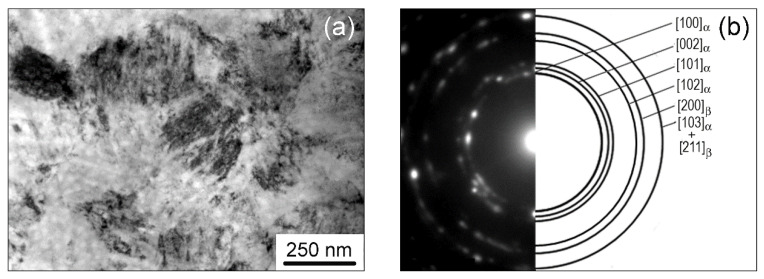

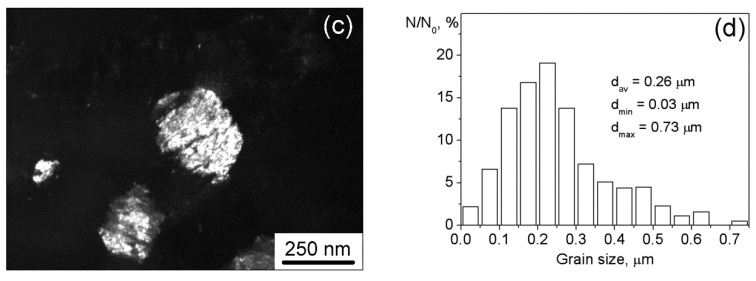
TEM images of the UFG Ti-2.9Al-4.5V-4.8Mo-0.15H alloy: (**a**) bright-field image and (**b**) corresponding SAED; (**c**) dark-field image in the reflection of (002)_α_ type; (**d**) size distribution of grain–subgrain elements.

In the Ti-2.9Al-4.5V-4.8Mo-0.15H alloy, after pressing, a uniform grain–subgrain UFG structure was also observed. Electron microscopic images of this structure are demonstrated in Figure 3. The value of average size (d_av_) of the UFG grain–subgrain structure elements in the Ti-2.9Al-4.5V-4.8Mo-0.15H alloy, as determined from the dark-field image (Figure 3c), was 0.26 ± 0.15 µm. The main bulk of the UFG Ti-2.9Al-4.5V-4.8Mo-0.15H alloy volume, similar to the UFG Ti-2.9Al-4.5V-4.8Mo alloy, was occupied by structural elements with sizes that varied from 0.1 to 0.3 μm (Figure 3d).

**Figure 4:**
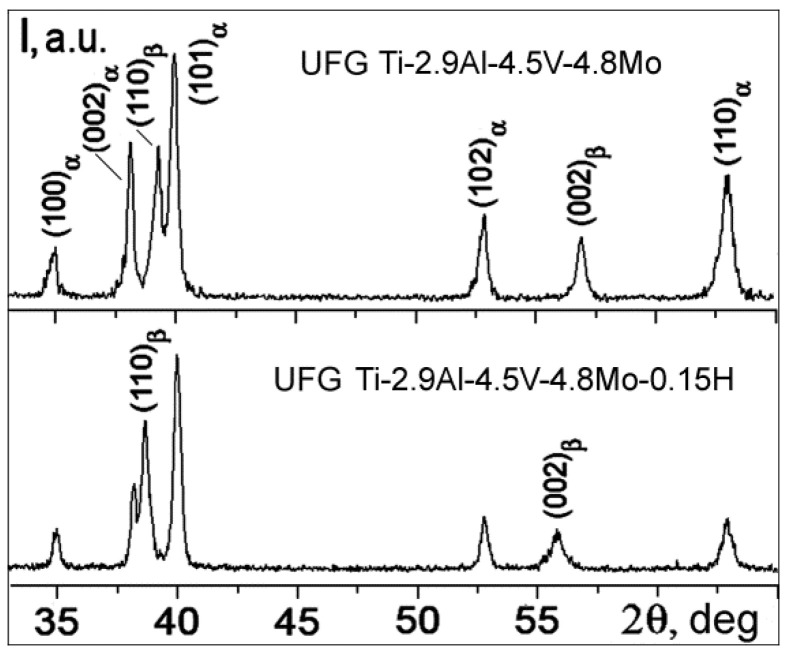
Diffraction patterns of the UFG titanium alloys.

X-ray structural analysis revealed that both of the UFG structures that formed as a result of pressing in Ti-2.9Al-4.5V-4.8Mo and Ti-2.9Al-4.5V-4.8Mo-0.15H alloys were two-phase (α + β) (Figure 4). In addition, in the hydrogenated FG and UFG Ti-2.9Al-4.5V-4.8Mo-0.15H alloys, there were no hydride precipitates, and all hydrogen was in a solid solution.

The results of X-ray diffraction studies of the structural and phase states of the FG and UFG Ti-2.9Al-4.5V-4.8Mo and Ti-2.9Al-4.5V-4.8Mo-0.15H alloys are presented in Table 1. It can be clearly observed that the formation of the UFG structure slightly changed the contents of the β-phase in Ti-2.9Al-4.5V-4.8Mo and Ti-2.9Al-4.5V-4.8Mo-0.15H alloys. It should be noticed that this value in the Ti-2.9Al-4.5V-4.8Mo-0.15H alloy was 7–8% higher in both states, compared to the appropriate states of the Ti-2.9Al-4.5V-4.8Mo alloy. This is apparently due to the hydrogen presence, which is known to be a stabilizer of the titanium β phase [16]. Additionally, as a result of hydrogenation, an increase in the lattice parameter of the β phase was observed; this indicates a reduction in the concentration of alloying elements such as vanadium and molybdenum in its volume [42]. Hydrogenation also leads to a rise in the values of internal stresses (σ) and dislocation density (ρ). Furthermore, the values of ρ and σ in the UFG Ti-2.9Al-4.5V-4.8Mo and Ti-2.9Al-4.5V-4.8Mo-0.15H alloys differed insignificantly from the similar values for the corresponding FG states. The ρ values in the β phase in the Ti-2.9Al-4.5V-4.8Mo and Ti-2.9Al-4.5V-4.8Mo-0.15H alloys in both states were 6–10 times, and the value of σ was ~2 times less than the corresponding values for the α phase.

### 3.2. Mechanical Properties

**Figure 5:**
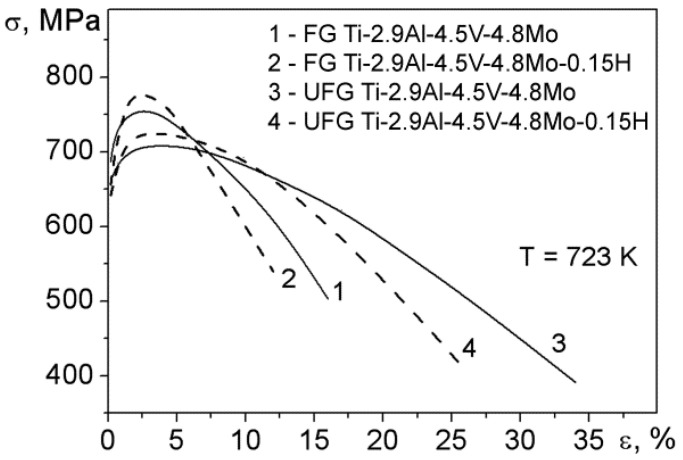
Tension curves of the FG and UFG Ti-2.9Al-4.5V-4.8Mo and Ti-2.9Al-4.5V-4.8Mo-0.15H alloys at 723 K.

The typical tension curves for the Ti-2.9Al-4.5V-4.8Mo and Ti-2.9Al-4.5V-4.8Mo-0.15H alloys at the temperature of 723 K are demonstrated in Figure 5. It can be clearly observed that the formation of the UFG structure and hydrogenation did not change the appearance of the tension curves. The tension curves of the Ti-2.9Al-4.5V-4.8Mo and Ti-2.9Al-4.5V-4.8Mo-0.15H alloys in both states had a short hardening stage, as well as a prolonged stage of declining stress. In this case, the value of the hardening effect for the Ti-2.9Al-4.5V-4.8Mo-0.15H alloy in both states was higher, compared to the corresponding states for the Ti-2.9Al-4.5V-4.8Mo alloy. It was shown above (Table 1) that the values of internal stresses and dislocation density in the Ti-2.9Al-4.5V-4.8Mo-0.15H alloy in both states were higher than in the Ti-2.9Al-4.5V-4.8Mo alloy. Therefore, the detected increase in the hardening effect of the Ti-2.9Al-4.5V-4.8Mo-0.15H alloy in both states, in comparison with the Ti-2.9Al-4.5V-4.8Mo alloy, indicates the facilitated nucleation and increased mobility of dislocations under deformation in the presence of hydrogen.

Table 2 presents the numerical values of the yield strength (σ_02_) and ultimate strength (σ_B_) that were calculated from the tension curves (Figure 5), as well as the value of deformation to failure (δ) of the Ti-2.9Al-4.5V-4.8Mo and Ti-2.9Al-4.5V-4.8Mo-0.15H alloys in both states at 723 K. It is seen that the values of σ_02_ and σ_B_ of the alloys differ from each other by less than 10%. The refinement of the grain size to an ultrafine one leads to an approximately two-fold growth in the δ value for both types of studied alloys. In this case, the δ values for hydrogenated alloys in both states were ~1.3–1.4 times lower than the corresponding values for non-hydrogenated alloys.

### 3.3. Creep

Creep tests have revealed a decrease in creep resistance of the Ti-2.9Al-4.5V-4.8Mo and Ti-2.9Al-4.5V-4.8Mo-0.15H alloys, which was caused by the UFG structure formation. Thus, at the used temperature, the rates of steady-state creep at an interval of (10^−7^ ÷ 10^−6^) s^−1^ for the Ti-2.9Al-4.5V-4.8Mo and Ti-2.9Al-4.5V-4.8Mo-0.15H alloys in the FG state are observed at stresses of (0.7–0.85)σ_02_. At the same time, similar values of the steady-state creep rates for these alloys in the UFG state are already evidenced at stresses of (0.2–0.3)σ_02_. The measurements of the hydrogen content in the samples of the FG and UFG Ti-2.9Al-4.5V-4.8Mo-0.15H alloys after creep have shown that, in the process of short-term (10 h) creep, the hydrogen concentration increased in the deformed part of the specimen and decreased in the undeformed part. Thus, during creep at a stress of 500 MPa, the hydrogen concentration on the working part of the Ti-2.9Al-4.5V-4.8Mo-0.15H alloy sample in the FG state increased from ~0.15 to ~0.18 wt %. On the non-deformable part of the sample, the hydrogen concentration decreased to ~0.13 wt. %. For the UFG Ti-2.9Al-4.5V-4.8Mo-0.15H alloy, the concentration of hydrogen on the sample’s working part increased to ~0.165 wt. %, and on the non-deformable part of the sample, it decreased to ~0.142 wt.%. Consequently, the assessment of the possibility of hydrogen degassing from the alloy during creep is difficult. However, additional studies of changes in the hydrogen concentration in the alloy upon annealing for 10 h in a vacuum at 723 K have demonstrated that hydrogen degassing does not occur under these conditions.

**Figure 6:**
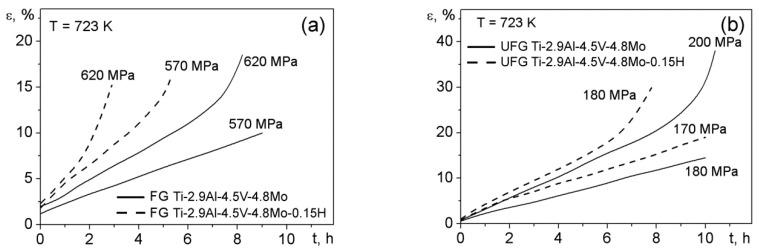
Creep curves: (**a**) FG Ti-2.9Al-4.5V-4.8Mo and Ti-2.9Al-4.5V-4.8Mo-0.15H alloys and (**b**) UFG Ti-2.9Al-4.5V-4.8Mo and Ti-2.9Al-4.5V-4.8Mo-0.15H alloys obtained at 723 K.

Figure 6 shows, as an example, the creep curves for the samples of the Ti-2.9Al-4.5V-4.8Mo and Ti-2.9Al-4.5V-4.8Mo-0.15H alloys at stresses of 570 and 610 MPa for the FG state (Figure 6a) and 170 and 200 MPa for the UFG state (Figure 6b). The presence of instantaneous deformation is a characteristic of all creep curves under the conditions studied. In addition, the instantaneous deformation of the Ti-2.9Al-4.5V-4.8Mo-0.15H alloy in both states exceeds the appropriate values of the Ti-2.9Al-4.5V-4.8Mo alloy by a factor of 1.5–2. The creep curves of the Ti-2.9Al-4.5V-4.8Mo alloy specimens in the FG and UFG states exhibited only two stages: steady-state and accelerated creep. On the creep curves of the Ti-2.9Al-4.5V-4.8Mo-0.15H alloy in both states, in addition to the two indicated stages, the stage of unsteady creep was also observed. In this case, the duration (concerning time and deformation) of the steady-state and accelerated creep stages of the FG and UFG Ti-2.9Al-4.5V-4.8Mo-0.15H alloys decreased, as compared to the appropriate states of Ti-2.9Al-4.5V-4.8Mo alloy. From a comparison of the creep curves of the Ti-2.9Al-4.5V-4.8Mo and Ti-2.9Al-4.5V-4.8Mo-0.15H alloys in the corresponding states, it was established that, for the studied interval of rates, the hydrogenation of the investigated titanium alloy resulted in a rise in the steady-state rates of creep by 1.3–2.5 times.

**Figure 7:**
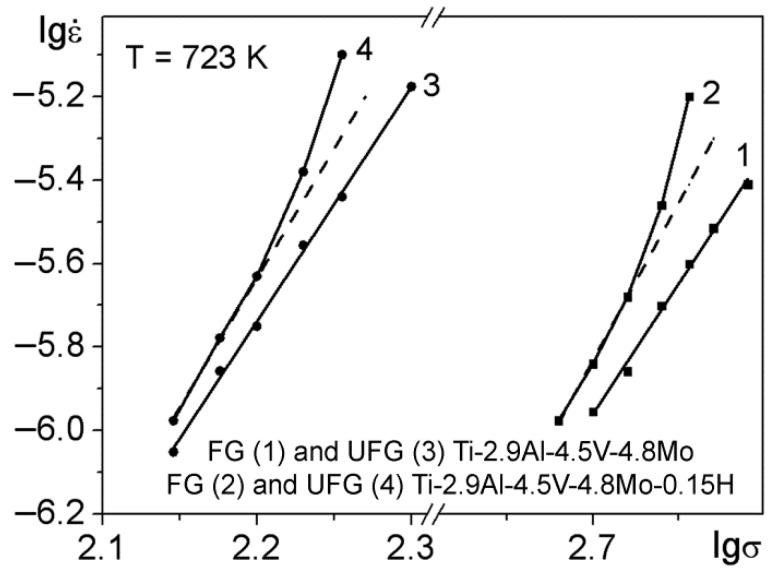
Experimental dependences of the steady creep rate on the stress of the FG and UFG titanium alloys.

The dependences of $\mathrm{lg}\dot{\varepsilon }=f(\mathrm{lg}\sigma )$ (where $\mathrm{lg}\dot{\varepsilon }$ is the logarithm of steady-state creep rate and lgσ is the logarithm of the applied stress) for the investigated alloys in the FG and UFG states are demonstrated in Figure 7. It can be seen from the figure that, under the test conditions used, the dependences for both states of the Ti-2.9Al-4.5V-4.8Mo alloy were close to linear. Consequently, it can be concluded that the creep for both states of this unhydrogenated alloy obeys the well-known creep power law [40]:

(5) $$\dot{\varepsilon }=A\sigma^{n}\exp(Q_{c}/RT),$$

where $\dot{\varepsilon }$ is the steady creep rate, *n* is the index of stress sensitivity to the creep rate, *A* is a constant, *Q_c_* is the effective creep activation energy, *R* is the universal gas constant, and *T* is the absolute temperature.

It follows from the slopes of the curves of the steady creep rate $\dot{\varepsilon }$ on the applied stress in the double-logarithmic coordinates (Figure 7) that the stress sensitivity indices *n* were equal to 6.3 and 5.6 for the Ti-2.9Al-4.5V-4.8Mo alloys in the FG and UFG states, respectively. The high values of the *n* index for the Ti-2.9Al-4.5V-4.8Mo alloy are consistent with the data of creep investigation for other titanium alloys that were under conditions similar to the applied test conditions [43,44,45,46,47]. The obtained values of the stress sensitivity index *n* indicate that the creep of the titanium alloy in both states and under the studied conditions was realized by the movement (glide + climb) of dislocations [40]. In this case, the steady-state creep rate was controlled by the dislocation climb [48].

The hydrogen presence in the solid solution of the Ti-2.9Al-4.5V-4.8Mo-0.15H alloy in the FG and UFG states leads to a violation of the creep power law (Figure 7). The values of the *n* index of the Ti-2.9Al-4.5V-4.8Mo-0.15H alloy in the FG and UFG states increased with a rise in the applied stress. In this case, in the entire stress range used, the values of the index *n* of the Ti-2.9Al-4.5V-4.8Mo-0.15H alloy in both states exceeded the relevant values of the Ti-2.9Al-4.5V-4.8Mo alloy (Figure 7).

Table 3 shows the *Q_c_* values of the Ti-2.9Al-4.5V-4.8Mo and Ti-2.9Al-4.5V-4.8Mo-0.15H alloys in the FG and UFG states, as determined by a temperature rise of 10 K and calculation using equation (4). Here, for comparison, the values of *Q_c_* are given, as observed in [45,46,47] for the creep of titanium alloys under conditions close to those used in this work. It can be realized from the data in the table that the *Q_c_* values obtained during the creep of the Ti-2.9Al-4.5V-4.8Mo and Ti-2.9Al-4.5V-4.8Mo-0.15H alloys were comparable in value with the *Q_c_* values for other titanium alloys, the main deformation mechanism of which was the dislocation motion controlled by the bulk diffusion of the titanium [45,46,47]. The formation of the UFG structure somewhat reduced the *Q_c_* value for both states of the studied alloys. Hydrogenation led to a rise in the value of *Q_c_*. In this case, the *Q_c_* value of the Ti-2.9Al-4.5V-4.8Mo-0.15H alloy in both states grew with an increase in the applied stress (Table 3).

### 3.4. Fracture

**Figure 8:**
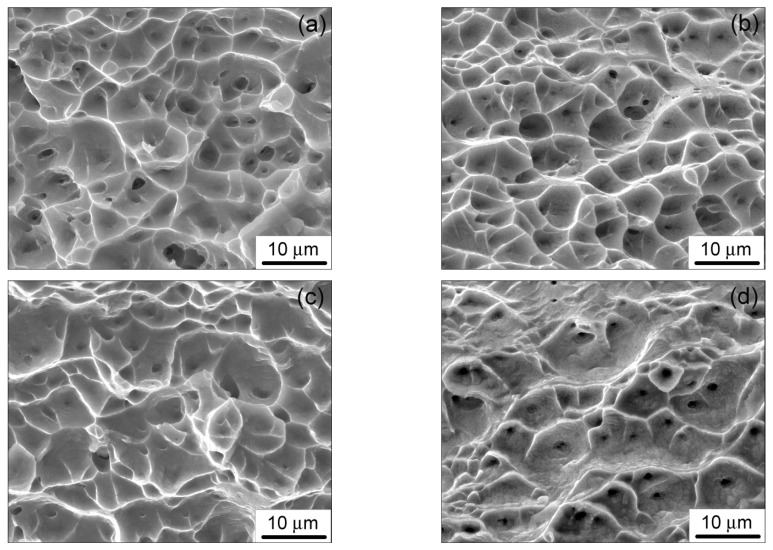
Fracture surface of the titanium alloys after creep at 723 K: (**a**) FG and (**b**) UFG Ti-2.9Al-4.5V-4.8Mo-0.15H; (**c**) FG and (**d**) UFG Ti-2.9Al-4.5V-4.8Mo.

The fracture of the Ti-2.9Al-4.5V-4.8Mo and Ti-2.9Al-4.5V-4.8Mo-0.15H alloys in both states occurred due to the formation of a neck and the subsequent separation along a plane almost perpendicular to the direction of the applied load. The typical fracture surfaces for the Ti-2.9Al-4.5V-4.8Mo and Ti-2.9Al-4.5V-4.8Mo-0.15H alloys are shown in Figure 8. It can be seen that the fracture surface of the alloys was uniform and had the form of a viscous dimple fracture. A common characteristic feature of the fracture surfaces of the alloys studied is the presence of pores. The pores are evenly distributed over the entire fracture surface and have a rounded shape, which indicates a significant contribution of the diffusion mechanism to their development. The pore density in the Ti-2.9Al-4.5V-4.8Mo-0.15H alloy in both states was noticeably higher, compared to the corresponding states of the Ti-2.9Al-4.5V-4.8Mo alloy. Cracks on the fracture surface of the alloys were not observed. Therefore, it can be assumed that the fracture of the Ti-2.9Al-4.5V-4.8Mo and Ti-2.9Al-4.5V-4.8Mo-0.15H alloys during creep under the studied conditions occurred through the formation and coalescence of pores.

## 4. Discussion

Structural studies have shown that hydrogenation does not change the structure morphology of the Ti-2.9Al-4.5V-4.8Mo alloy; instead, it leads to an increase in the volume fraction of the β phase. A rise in the volume fraction of the β phase is observed in titanium alloys upon hydrogenation in the (α + β) region [16,17]. This effect is caused by the fact that hydrogen is a stabilizer of the β phase, and it reduces the temperature of α→β phase transformation [17,49]. At the same time, there are data in the literature that suggest that the broadening of the α/β boundaries occurs as a result of the hydrogenation of the alloy of the Ti-Al-V system [31]. The broadening of the α/β boundaries, according to [31], is a consequence of the formation of a β phase that is depleted in the alloying elements at the grain boundaries. The absence of such broadening in the alloy studied after hydrogenation is apparently due to the thin lamellar structure of the alloy and performed stabilizing annealing at 673 K for 30 h. As a result of such annealing, the alloying of the β phase can become homogeneous. However, the concentration of alloying elements in the β phase will be lower, compared to the non-hydrogenated state. This will lead to an increase in its lattice parameter [42]. The presence of hydrogen in the β phase also increases its lattice parameter [50].

The UFG structure observed in the Ti-2.9Al-4.5V-4.8Mo and Ti-2.9Al-4.5V-4.8Mo-0.15H alloys is the typical one that is formed in (α + β) titanium alloys by the methods of severe plastic deformation (SPD). The structure after SPD is characterized by high density of dislocations (up to 10^15^ m^2^) and crystal lattice microdistortions (up to 10^−2^) [11,12,13]. Performed stabilizing annealing reduces the dislocation density in the Ti-2.9Al-4.5V-4.8Mo and Ti-2.9Al-4.5V-4.8Mo-0.15H alloys in the UFG state to a level that is close to the FG state.

After stabilizing annealing, the strength characteristics at room temperature for the Ti-2.9Al-4.5V-4.8Mo and Ti-2.9Al-4.5V-4.8Mo-0.15H alloys in the UFG state are approximately 40% (up to 1400 MPa) higher, compared to FG state (up to 1000 MPa), while maintaining plasticity at the level of (12–13%) [14]. The titanium alloys of the Ti-Al-V system in the UFG state have similar strength characteristics. However, their plasticity does not exceed 8% [11,12,13]. With an increase in the test temperature, the strength characteristics of the Ti-2.9Al-4.5V-4.8Mo and Ti-2.9Al-4.5V-4.8Mo-0.15H alloys in the UFG state decrease, while the plasticity increases at a faster rate, compared to the FG state. In the temperature range of 873–923 K, the plasticity of the Ti-2.9Al-4.5V-4.8Mo and Ti-2.9Al-4.5V-4.8Mo-0.15H alloys in the UFG state reaches ~700 and ~400%, respectively [14]. The plasticity of FG alloys in this temperature range does not exceed 70%.

The results presented above indicate that the formation of a stabilized UFG structure in the Ti-2.9Al-4.5V-4.8Mo alloy results in a decrease in its creep resistance. This is consistent with the results of the creep studies of UFG titanium alloys of the Ti–6Al–4V system [45,46]. It was noted above that the observed values of the *n* index and *Q_c_* of the Ti-2.9Al-4.5V-4.8Mo alloy are characteristic of titanium alloys, the main creep mechanism of which is the dislocation motion, which is controlled by the bulk diffusion of titanium. A reduction in the UFG Ti-2.9Al-4.5V-4.8Mo alloy’s resistance to creep can be a consequence of the development of deformation mechanisms, such as sliding along grain boundaries (GBS) and sliding along boundaries of grain conglomerate (cooperative GBS) during creep. This is indicated by a decrease in the values of the index *n* and *Q_c_* of the Ti-2.9Al-4.5V-4.8Mo alloy in the UFG state, in comparison with the FG state (Figure 7 and Table 3). A significant contribution of GBS and cooperative GBS to the deformation of UFG materials in the range of moderate temperatures (*T* < 0.4*T_melt_*) was observed in [51,52,53,54]. However, in order to confirm the assumption regarding the GBS development in the alloy studied and conditions used, it is necessary to carry out special microstructural studies. Performed investigations have demonstrated that the hydrogen present in the Ti-2.9Al-4.5V-4.8Mo-0.15H alloy in both states results in a rise in the steady-state creep rate, as well as a drop in the time and deformation to failure, as compared to the relevant states of the Ti-2.9Al-4.5V-4.8Mo alloy. The increase in the steady-state creep rate of the Ti-2.9Al-4.5V-4.8Mo-0.15H alloy in both states, compared to the corresponding states of the Ti-2.9Al-4.5V-4.8Mo alloy, can be associated with facilitated nucleation and an increase in the mobility of dislocations in the presence of hydrogen in the solid solution [23,24,25,26]. This assumption was confirmed by the growth in the *Q_c_* values of the Ti-2.9Al-4.5V-4.8Mo-0.15H alloy in both states, in comparison with the corresponding *Q_c_* values of the Ti-2.9Al-4.5V-4.8Mo alloy (Table 3). In addition, a rise in the volume fraction of the β phase in the Ti-2.9Al-4.5V-4.8Mo-0.15H alloy during hydrogenation (Table 1) can also contribute to an increase in the steady-state creep rate. This is due to the fact that, at a temperature of 723 K, the coefficient of the bulk diffusion of titanium, which controls the dislocation climbing, in the β phase was approximately two orders of magnitude higher than it was in the α phase [40].

It should be noted that there are data in the literature that demonstrate that a decrease in the steady-state creep rate is observed in titanium alloys when hydrogen is in the alloy of a solid solution. For example, in works [29,30], a decrease in the rate of steady-state creep under the specified test conditions, when studying the creep of titanium alloys of the Ti-Al-V-H system at the temperature interval (723–923) K, was recorded. Simultaneously, it was found in [31] that hydrogen’s impact on the steady-state creep rate of the Ti-Al-V-H system alloys at temperatures of 873 and 923 K was determined by the dissolved hydrogen content in the material. For example, when the concentration of dissolved hydrogen was equal to 0.2%, the steady-state creep rate of the Ti-Al-V-H alloy practically did not change; at the concentration of 0.4% dissolved hydrogen, it decreased. The observed contradictory impact of the dissolved hydrogen on the steady-state creep rate of titanium alloys can apparently be explained by the solid solution hardening of the β phase, which can contribute significantly to the alloy’s resistance to creep in the case of a high hydrogen concentration. For example, in [30], the volume fraction of the β phase in the Ti-Al-V-H alloy did not exceed 14 vol.%, and the hydrogen concentration was 0.23%. In the present work, in the studied Ti-2.9Al-4.5V-4.8Mo-0.15H alloy, the volume fraction of the β-phase and hydrogen concentration were equal to 27–30 vol.% and 0.15%, respectively. This allows us to assume that there was an insignificant contribution of solid solution hardening of the β phase to the resistance of the Ti-2.9Al-4.5V-4.8Mo-0.15H alloy to creep.

The decrease in the duration of the steady-state creep stage and total deformation to failure of the Ti-2.9Al-4.5V-4.8Mo-0.15H alloy in the FG and UFG states, in comparison with the corresponding states of the Ti-2.9Al-4.5V-4.8Mo alloy, was more likely associated with hydrogen’s ability to redistribute in elastic stress fields, thus accumulating in the most stressed areas. In such regions, the formation of pores will occur, which contributes to the localization of plastic deformation, transition to an accelerated stage of creep, and premature fracture of the alloy. This assumption is consistent with the above indicated results of hydrogen content measurement, which show that the hydrogen concentration increases in the bulk of the deformable part of the Ti-2.9Al-4.5V-4.8Mo-0.15H alloy samples and decreases in the bulk of the non-deformable parts. In addition, the pore densities on the fracture surface of the Ti-2.9Al-4.5V-4.8Mo-0.15H alloy samples were higher, compared to the samples of Ti-2.9Al-4.5V-4.8Mo alloy (Figure 8). The pores have a rounded shape, which indicates a significant contribution of the diffusion mechanism to their development.

Violation of the creep power law for the Ti-2.9Al-4.5V-4.8Mo-0.15H alloy in both states under the conditions studied is apparently associated with the dependence of the diffusion mobility of hydrogen on the value of the applied stress. According to [55], as the applied tensile stress grows, the rate of hydrogen flux increases. Following a rise in the rate of hydrogen flux, the rate of motion of the dislocations interacting with hydrogen also increase.

It should be noted that the large difference in the stress values, at which the same steady-state creep rates were observed for the investigated FG and UFG alloys, do not allow, based on the results obtained, for unambiguously determining which of these states (FG or UFG) was more resistant to hydrogen embrittlement during creep under the conditions studied. The question regarding the resistance of UFG titanium alloys to hydrogen embrittlement can be clarified in future studies.

## 5. Conclusions

In this work, we studied the effect of hydrogen’s presence in a solid solution on the creep regularities of a titanium Ti-2.9Al-4.5V-4.8Mo alloy in the FG and UFG states at 723 K and interval rates of (10^−7^ ÷ 10^−6^) s^−1^.

The combination of the values of the stress sensitivity indices of the steady creep rate and effective creep activation energy indicated that the main deformation mechanism of the Ti-2.9Al-4.5V-4.8Mo alloy in both states under the creep was the dislocation climb controlled by the bulk self-diffusion of titanium.

The formation of an ultrafine-grained structure led to an increase in the steady-state creep rate of the Ti-2.9Al-4.5V-4.8Mo and Ti-2.9Al-4.5V-4.8Mo-0.15H alloys under the studied conditions. At the same time, the stress sensitivity of the steady-state creep rate of the UFG alloys was lower, compared to the FG alloys.

Hydrogenation of the Ti-2.9Al-4.5V-4.8Mo alloy in both states to 0.15 wt.% led to an increase in the steady-state creep rate and decrease in the time and value of deformation to failure. At the same time, the influence of hydrogen on the indicated creep parameters of the alloy in the UFG state was lower than it was in the FG state.

The dependence of the steady-state creep rate on the initial stress of the Ti-2.9Al-4.5V-4.8Mo alloy in both states at a temperature of 723 K was satisfactorily described by the creep power law. Hydrogenation of the alloy to 0.15 wt. % resulted in the violation of the creep power law. With an increase in the initial stress, the growth in the steady-state creep rate of the Ti-2.9Al-4.5V-4.8Mo-0.15H alloy in both states rose, and the strain at the stage of steady-state creep decreased.

The results obtained could be useful in the development of technologies for molding critical products of titanium alloys by the creep method.

## Figures and Tables

**Table 1:** Results of X-ray diffraction analysis for samples of the titanium alloys in different states.

State of Alloy	d, μm	Phase Composition ±1%	The β Phase Lattice Parameter, nm	ρ, m^−2^	σ, MPa
FG Ti-2.9Al-4.5V-4.8Mo	9	β = 20 α = 80	0.3238	β—2.3·10^12^ α—2.7·10^13^	β—115 α—254
FG Ti-2.9Al-4.5V-4.8Mo-0.15H	9	β = 27 α = 73	0.3259	β—1.1·10^13^ α—7.5·10^13^	β—159 α—310
UFG Ti-2.9Al-4.5V-4.8Mo	0.23	β = 22 α = 78	0.3246	β—3.5·10^12^ α—3.0·10^13^	Β—143 α—276
UFG Ti-2.9Al-4.5V-4.8Mo-0.15H	0.26	β = 30 α = 70	0.3265	β—1.5·10^13^ α—9.2·10^13^	β—191 α—378

**Table 2:** Mechanical properties of the Ti-2.9Al-4.5V-4.8Mo and Ti-2.9Al-4.5V-4.8Mo-0.15H alloys at a temperature of 723 K.

State of Alloy	σ_02_ ± 15, MPa	σ_B_ ± 15, MPa	δ ± 1%
FG Ti-2.9Al-4.5V-4.8Mo	687	755	16
FG Ti-2.9Al-4.5V-4.8Mo-0.15H	656	775	11
UFG Ti-2.9Al-4.5V-4.8Mo	661	707	34
UFG Ti-2.9Al-4.5V-4.8Mo-0.15H	642	729	26

**Table 3:** The effective creep activation energy for the Ti-2.9Al-4.5V-4.8Mo and Ti-2.9Al-4.5V-4.8Mo-0.15H alloys.

*Q_c_*, ± 20, kJ/mole				
FG Ti-2.9Al-4.5V-4.8Mo	FG Ti-2.9Al-4.5V-4.8Mo-0.15H	UFG Ti-2.9Al-4.5V-4.8Mo	UFG Ti-2.9Al-4.5V-4.8Mo-0.15H	Titanium alloys [45,46,47]
σ = 475–540 MPa		σ = 140–160 MPa		
321	349–372	255	289–325	289–375

## Data Availability

Not applicable.
